# Uncommon Clinical Presentations of Sporotrichosis: A Two-Case Report

**DOI:** 10.3390/pathogens10101249

**Published:** 2021-09-27

**Authors:** Erick Martínez-Herrera, Roberto Arenas, Rigoberto Hernández-Castro, María Guadalupe Frías-De-León, Carmen Rodríguez-Cerdeira

**Affiliations:** 1 Research Unit, Regional Hospital of High Specialty of Ixtapaluca, Ixtapaluca 56530, Mexico; erickmartinez_69@hotmail.com (E.M.-H.); mgfrias@hraei.gob.mx (M.G.F.-D.-L.); 2 Postgraduate Studies and Research Section, Higher School of Medicine, National Polytechnic Institute, Mexico City 11340, Mexico; 3 Efficiency, Quality, and Costs in Health Services Research Group (EFISALUD), Galicia Sur Health Research Institute (IIS Galicia Sur), SERGAS-UVIGO, 36213 Vigo, Spain; rarenas98@gmail.com; 4 Department of Mycology, General Hospital “Dr. Manuel Gea González”, Mexico City 14080, Mexico; 5 Department of Ecology of Pathogens, General Hospital “Dr. Manuel Gea González”, Mexico City 14080, Mexico; rigo37@gmail.com; 6 Department of Dermatology, Hospital Vithas Ntra. Sra. de Fátima and University of Vigo, 36206 Vigo, Spain; 7 Campus of Vigo, University of Vigo, 36310 Vigo, Spain

**Keywords:** sporotrichosis, *Sporothrix schenckii*, PCR, pathogen–host interaction, clinical manifestations

## Abstract

Sporotrichosis is a subcutaneous endemic mycosis caused by species of the *Sporothrix schenckii* complex. The most common clinical form of the disease is lymphocutaneous, while the fixed cutaneous and disseminated cutaneous forms are rare. Moreover, it is more prevalent in immunocompetent individuals. In this study, we present two cases of sporotrichosis with uncommon clinical forms: fixed cutaneous (Case 1) and disseminated cutaneous (Case 2). Both cases were diagnosed in immunocompetent males from endemic regions in Mexico, who had at least 1 year of evolution without improvement in response to prior nonspecific treatments. The diagnosis of sporotrichosis caused by *S. schenckii* sensu stricto was established through the isolation of the pathogen and its identification through the amplification of a 331 bp fragment of the gene encoding calmodulin. In both cases, improvement was observed after treatment with potassium iodide. Cases 1 and 2 illustrate the rarity of these clinical forms in individuals residing in endemic areas; hence, it is important to ensure a high index of clinical suspicion for the diagnosis of mycosis, as the differential diagnoses vary widely.

## 1. Introduction

Sporotrichosis is a subcutaneous, granulomatous, chronic fungal infection that occurs worldwide; however, most cases are reported in Mexico, Central and South America, and some areas of South Africa [1]. This form of mycosis is usually caused by the traumatic inoculation of species of the *Sporothrix schenckii* complex, which include six dimorphic and saprobic species: *S. albicans*, *S. brasiliensis*, *S. globosa*, *S. luriei*, *S. mexicana*, and *S. schenckii* sensu stricto [2,3,4]. These fungi present significant differences in their geographical distribution, biochemical properties, degree of virulence, and susceptibility to antifungals; hence, their specific identification in clinical cases is relevant [2,3,4,5]. These fungi enter the human body by traumatic inoculation and ascend through the lymphatic channels to develop granulomatous lesions and produce nodular ulcerative papules, which can also occur as fixed cutaneous, disseminated cutaneous, or systemic forms [2,6]. Several factors, such as the host’s immune status, the virulence of the fungus, and the site and depth of the trauma, can determine the different type of clinical presentation [7]. The most common clinical form of sporotrichosis is lymphocutaneous (70–95%) [6], followed by fixed cutaneous (>30%), which is observed in workers handling soil or decaying vegetal material, such as agriculturists, gardeners, and florists [7,8]. On the other hand, the disseminated cutaneous (8%) and extracutaneous (1%) forms are less common and mainly occur in persons with immunosuppression [9,10]. Clinical suspicion, along with the isolation and identification of the *Sporothrix* species from clinical samples, is vital for the diagnosis of any clinical form of sporotrichosis [1,2].

In this study, we report two cases of sporotrichosis with infrequent clinical forms identified in Mexico (fixed cutaneous and disseminated cutaneous); both cases were diagnosed in immunocompetent patients.

## 2. Case 1

**Figure 1:**
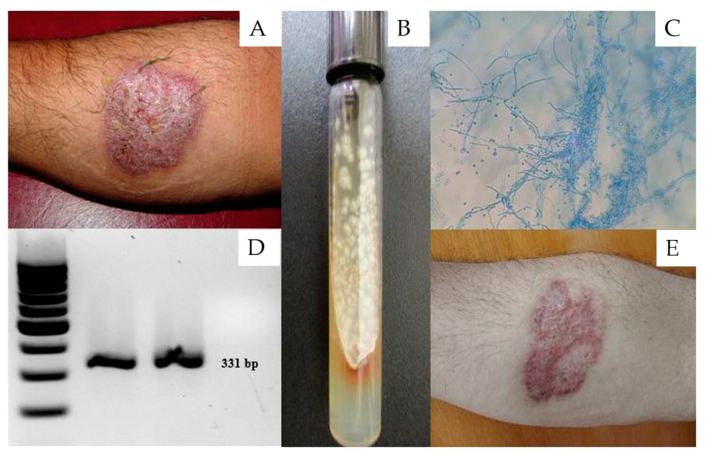
(**A**) Fixed cutaneous sporotrichosis. (**B**) Culture of the *Sporothrix schenckii* complex in Sabouraud dextrose agar. (**C**) Microscopic view of *Sporothrix schenckii.* (**D**) Molecular analysis (PCR) of *Sporothrix schenckii* sensu stricto. (**E**) Fixed cutaneous sporotrichosis cured via potassium iodide treatment.

A 61-year-old male agriculturist residing in Oaxaca, Mexico, attended a dermatological consultation due to the presence of a warty nodular plaque, 5 cm in diameter with well-defined edges, located in the anterior medial part of the right forearm (Figure 1A), and with a year of evolution. The patient did not present with comorbidities and reported receiving multiple empirical treatments prior to the consultation. Chromoblastomycosis or tuberculosis verrucous cutis was suspected. A direct examination of the plaque was performed using 40% KOH, followed by a biopsy and culturing in Sabouraud medium at 28 °C for 8 days. The first culture was negative. The histopathology revealed pseudoepitheliomatous hyperplasia and suppurative granuloma; no parasitic elements were observed after Periodic Acid-Schiff (PAS) and Gomori-Grocott staining. A colony with the typical macroscopic and microscopic morphology of the *S. schenckii* complex (Figure 1B,C) was isolated in the culture, thereby leading to a diagnosis of fixed cutaneous sporotrichosis. The identity of the pathogen was confirmed by the amplification of a 331 bp fragment (Figure 1D) of the *CAL* gene (calmodulin) using multiplex PCR [11]. The patient received oral treatment with 3 g potassium iodide (KI) daily for 4 months, resulting in a total dose of 500 g. The patient showed complete recovery, leaving only a residual scar (Figure 1E).

## 3. Case 2

**Figure 2:**
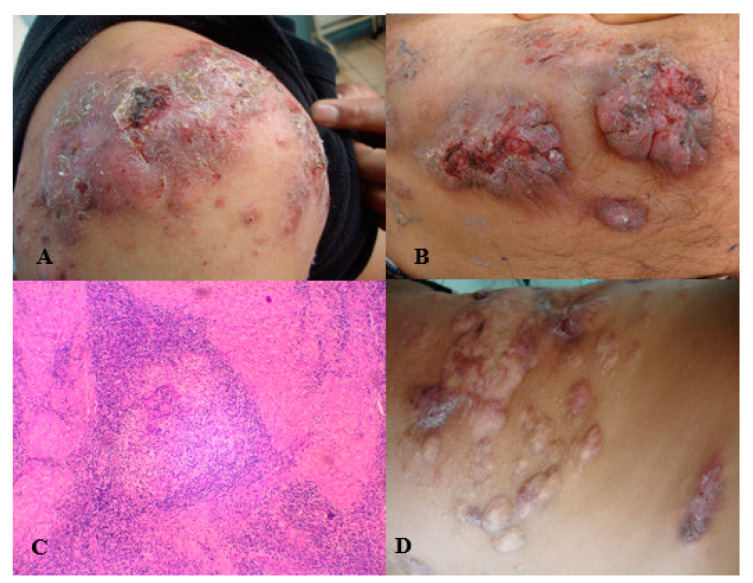
(**A**) Disseminated cutaneous sporotrichosis with nodular and ulcerated lesions. (**B**) Disseminated cutaneous sporotrichosis with verrucous lesions. (**C**) Suppurative granuloma (HE40x). (**D**) Improvement observed after 1 year of potassium iodide treatment.

A 21-year-old male patient residing in Guanajuato, Mexico, visited a medical consultation due to the presence of multiple nodular and warty plaques (some ulcerated and with scabs) that affected the anterior and posterior areas of his chest, abdominal wall, arms, and forearms (Figure 2A,B). The patient reported a 2-year evolution without healing after multiple empirical treatments. Tuberculosis verrucous cutis, chromoblastomycosis, and sporotrichosis were suspected. A skin test was performed with sporotrichin, followed by a biopsy and culturing in Sabouraud medium at 28 °C for 8 days. The skin test was positive, showing an induration > 5 mm in diameter. The main histopathological finding was suppurative granuloma; no fungal elements were found (Figure 2C). The growth of a colony morphologically compatible with *Sporothrix* spp. was observed in the culture. Identification of the *S. schenckii* sensu stricto species was carried out by amplification of a 331 bp fragment of the *CAL* gene [11], as described in Case 1. The patient received 3 g of KI daily for 12 months and showed a significant improvement. However, some injuries and active infection were still observed (Figure 2D); thus, the treatment was continued for 6 more months.

## 4. Discussion

Sporotrichosis is the most common subcutaneous mycosis in Mexico, with the lymphocutaneous form being the most common, while the fixed and disseminated cutaneous forms are rare [1,6]. Here, we report one case each of fixed cutaneous sporotrichosis (Case 1) and disseminated cutaneous sporotrichosis (Case 2) in patients from Oaxaca and Guanajuato, respectively, which are regions of Mexico with a high incidence of sporotrichosis [1].

Fixed cutaneous sporotrichosis usually occurs at the inoculation site itself [8]. Injuries are asymptomatic and erythematous, presenting in the form of nodules or warty plaques and occasionally ulcers that do not heal or have small abscesses. The lesions may resemble keratoacanthoma, facial cellulite, gangrenous pyoderma, nodular prurigo, soft tissue sarcoma, basal cell carcinoma, erysipeloid, or rosacea [1,2]. This clinical presentation usually occurs in immunocompetent individuals, in whom minimal lesions may disappear spontaneously or persist if no treatment is provided [2]. Appropriate drug treatment provides a good prognosis [12].

In Case 1, this patient with a fixed cutaneous sporotrichosis diagnosis offered several points of clinical interest, such as the low-frequency clinical form; the difficulty of demonstrating the presence of fungal cells in the biopsy, which usually complicates the diagnosis [13]; and the importance of considering cutaneous sporotrichosis in the differential diagnosis of skin nodules, especially when the patient does not respond to first-line therapies. In this case, the patient reported receiving treatments for bacterial infections without a favorable result, demonstrating that the clinical suspicion of mycosis or even sporotrichosis was non-existent, even though the patient resided in an endemic area and engaged in activities considered to be risky for contracting a fungal infection, i.e., agriculture. In addition, the warty clinical form of the disease made *t*uberculosis verrucous cutis or chromoblastomycosis a more likely possibility.

On the other hand, disseminated cutaneous sporotrichosis is a rare form typically observed in patients immunocompromised by HIV or with iatrogenic immunosuppression; infections may be lethal for such patients [14,15,16,17]. There are few reported cases that involve immunocompetent people [18]. The clinical manifestations of disseminated cutaneous sporotrichosis include ulcerated nodules and warty plaques [19].

In Case 2, the diagnosis was disseminated cutaneous sporotrichosis; however, the patient showed no sign of immunocompromise. Although the patient responded favourably to potassium iodide (KI) treatment for 12 months, it was necessary to prolong the treatment for effective resolution of the disease. This may have been due to differences in the virulence characteristics and antifungal susceptibility among species and even isolates [2,3,4]. We attributed this dissemination to the non-specific antibiotic treatment received by the patient prior to diagnosis. This case illustrates the rarity of clinical presentation in immunocompetent individuals, where it is vital to have a high degree of clinical suspicion for diagnosing sporotrichosis if the patient resides in endemic areas, as differential diagnoses can vary widely [1].

Both cases (1 and 2) indicate that clinicians should consider the possibility of the fixed or disseminated cutaneous form of sporotrichosis, even though the lymphocutaneous form is the most prevalent in immunocompetent patients. Similarly, clinicians should be aware that at least six species of fungi can cause mycosis and that some of these species are more virulent, such as *S. brasiliensis* [20]. The gold standard for diagnosing sporotrichosis is microscopic characterisation of the morphology of the isolated pathogen in culture; however, as the phenotypic characteristics of *Sporothrix* spp. are similar, it is currently necessary to use molecular methods for species-level identification [11]. In addition, the identification of *Sporothrix* species has relevant implications for selection of the antifungal therapy, as it has been observed that the response to antifungals in vitro varies among *S. brasiliensis*, *S. schenckii*, *S. globosa*, and *S. Mexicana* [11].

In the present study, *S. schenckii* sensu stricto was identified through a multiplex PCR trial that allowed the differentiation of species of the *Sporothrix* complex [11]. The multiplex PCR method has a low cost and is practically accessible for any microbiological diagnostic laboratory. In both cases, the response to KI treatment was adequate.

In conclusion, the cases presented here reveal that patients often overlook painless skin lesions, which are often treated with empirical treatments. This can, in turn, lead to a more severe manifestation of the disease. Therefore, health authorities should provide more accessible health services in areas where subcutaneous mycosis is common.

## Data Availability

The datasets used and/or analysed during the present study are available from the corresponding author on reasonable request.

## Abbreviations

Polymerase chain reaction (PCR); Human immunodeficiency virus (HIV).
